# Heterogeneous Infectivity and Pathogenesis of SARS-CoV-2 Variants Beta, Delta and Omicron in Transgenic K18-hACE2 and Wildtype Mice

**DOI:** 10.3389/fmicb.2022.840757

**Published:** 2022-05-04

**Authors:** Ferran Tarrés-Freixas, Benjamin Trinité, Anna Pons-Grífols, Miguel Romero-Durana, Eva Riveira-Muñoz, Carlos Ávila-Nieto, Mónica Pérez, Edurne Garcia-Vidal, Daniel Perez-Zsolt, Jordana Muñoz-Basagoiti, Dàlia Raïch-Regué, Nuria Izquierdo-Useros, Cristina Andrés, Andrés Antón, Tomàs Pumarola, Ignacio Blanco, Marc Noguera-Julián, Victor Guallar, Rosalba Lepore, Alfonso Valencia, Victor Urrea, Júlia Vergara-Alert, Bonaventura Clotet, Ester Ballana, Jorge Carrillo, Joaquim Segalés, Julià Blanco

**Affiliations:** ^1^IrsiCaixa AIDS Research Institute, Can Ruti Campus, UAB, Badalona, Spain; ^2^Barcelona Supercomputing Center, Barcelona, Spain; ^3^Unitat mixta d’investigació IRTA-UAB en Sanitat Animal, Centre de Recerca en Sanitat Animal (CReSA), Campus de la Universitat Autònoma de Barcelona (UAB), Bellaterra, Spain; ^4^IRTA, Programa de Sanitat Animal, Centre de Recerca en Sanitat Animal (CReSA), Campus de la Universitat Autònoma de Barcelona (UAB), Bellaterra, Spain; ^5^Germans Trias i Pujol Research Institute (IGTP), Badalona, Spain; ^6^CIBER Enfermedades Infecciosas (CIBERINFEC), Instituto de Salud Carlos III, Madrid, Spain; ^7^Respiratory Virus Unit, Department of Microbiology, Vall d’Hebron Institut de Recerca (VHIR), Vall d’Hebron Hospital Universitari, Vall d’Hebron Barcelona Hospital Campus, Barcelona, Spain; ^8^Germans Trias i Pujol Hospital, Badalona, Spain; ^9^University of Vic–Central University of Catalonia (UVic-UCC), Vic, Spain; ^10^Catalan Institution for Research and Advanced Studies (ICREA), Barcelona, Spain; ^11^Departament de Sanitat i Anatomia Animals, Facultat de Veterinària, Universitat Autònoma de Barcelona (UAB), Campus de la UAB, Bellaterra, Spain

**Keywords:** SARS-CoV-2 variants of concern, ACE2, viral load, histology, *in silico* modeling, infection, K18-hACE2 mice, wildtype mice

## Abstract

The emerging SARS-CoV-2 variants of concern (VOCs) may display enhanced transmissibility, more severity and/or immune evasion; however, the pathogenesis of these new VOCs in experimental SARS-CoV-2 models or the potential infection of other animal species is not completely understood. Here we infected K18-hACE2 transgenic mice with B.1, B.1.351/Beta, B.1.617.2/Delta and BA.1.1/Omicron isolates and demonstrated heterogeneous infectivity and pathogenesis. B.1.351/Beta variant was the most pathogenic, while BA.1.1/Omicron led to lower viral RNA in the absence of major visible clinical signs. In parallel, we infected wildtype (WT) mice and confirmed that, contrary to B.1 and B.1.617.2/Delta, B.1.351/Beta and BA.1.1/Omicron can infect them. Infection in WT mice coursed without major clinical signs and viral RNA was transient and undetectable in the lungs by day 7 post-infection. *In silico* modeling supported these findings by predicting B.1.351/Beta receptor binding domain (RBD) mutations result in an increased affinity for both human and murine ACE2 receptors, while BA.1/Omicron RBD mutations only show increased affinity for murine ACE2.

## Introduction

Severe Acute Respiratory Syndrome Coronavirus 2 (SARS-CoV-2) is a virus of zoonotic origin responsible for the Coronavirus Disease 2019 (COVID-19) that emerged by the end of 2019 in Wuhan, China (Holmes et al., 2021; Rasmussen, 2021). From there, it rapidly spread across the world fueling an unprecedented pandemic. Throughout the pandemics, the emergence of new SARS-CoV-2 variants has raised awareness that these could potentially be more infectious and/or could evade immune responses elicited by previous infection or vaccination (Trinité et al., 2021a; Wang et al., 2021a).

To date, mass genotyping has prompted the identification of several variants of concern (VOC; Mullen et al., 2020; Wang et al., 2021b), of note: the B.1.1.7/Alpha VOC —first described in the United Kingdom— showed higher transmissibility; the B.1.351/Beta and P.1/Gamma variants (initially described in South Africa and Brazil, respectively) demonstrated increased resistance to neutralizing antibodies elicited by other variants and/or vaccination (Dejnirattisai et al., 2021; Planas et al., 2021). Subsequently, the B.1.617.2/Delta variant, identified in India, rapidly supplanted all other described SARS-CoV-2 variants at the time, owing to its enhanced transmissibility (Dhar et al., 2021; Parums, 2021). In turn, B.1.617.2/Delta and subvariants were completely displaced by themore recent, and highly mutated B.1.1.529/Omicron variant, which arose in sub-Saharan Africa (Karim and Karim, 2021; Allen et al., 2022; Lambrou et al., 2022).

B.1.351/Beta’s greater resistance to neutralization was especially concerning since it could potentially impact on vaccination programs (Shen et al., 2021) however, worldwide spread was minimal. Furthermore, the B.1.617.2/Delta VOC showed a combination of both higher transmissibility and slight resistance to neutralization (Li et al., 2021). Mutations in the Spike (S) protein are responsible for these phenotypes by modifying neutralizing epitopes and/or increasing the affinity for the human angiotensin converting enzyme 2 (hACE2) receptor (Gan et al., 2021). In that sense, B.1.1.529/Omicron displays an unprecedented accumulation of mutations in the S protein, some of which were previously described in other variants and have been associated with increased transmissibility and antibody evasion (Petersen et al., 2021; Andrews et al., 2022; Grabowski et al., 2022; Liu et al., 2022; Planas et al., 2022). Despite showing high transmissibility and the strongest immune evasion yet, this variant could be less pathogenic (Iuliano et al., 2022). In addition, mutations can also modify the susceptibility of other host species to the virus, therefore potentially broadening the animal reservoir scope (Conceicao et al., 2020; Liu et al., 2021).

Animals are well-known coronavirus reservoirs (Menachery et al., 2017). While the intermediate host species of SARS-CoV-2 remains elusive, transmission from humans to domestic, farm, and zoo animals has been documented (Muñoz-Fontela et al., 2020; Oreshkova et al., 2020; Segalés et al., 2020). The landscape of susceptible species is also governed by S protein mutations, which can modulate the affinity to animal ACE2 (Garry, 2021). Notably, it has already been reported that new variants such as B.1.351/Beta and B.1.1.529/Omicron can infect SARS-CoV-2 resistant species like wildtype (WT) mice (Kant et al., 2021; Montagutelli et al., 2021; Shuai et al., 2021; Zhang et al., 2022). However, a complete *in vivo* susceptibility analysis has not yet been carried out.

The resistance of WT mice to ancestral SARS-CoV-2 infection made transgenic hACE2 mice one of the main experimental models for the *in vivo* study of novel vaccines and treatments (Bao et al., 2020; Jiang et al., 2020), together with golden Syrian hamsters (Imai et al., 2020; Choudhary et al., 2022). The K18-hACE2 mouse strain, a model used for SARS-CoV studies, is susceptible to SARS-CoV-2 infection and develops a severe disease. In these mice the hACE2 transgene is driven by the cytokeratin-18 (K18) gene promoter and hence it is expressed in many tissues, like lungs, intestines, and brain (Zheng et al., 2021). Widespread hACE2 expression in K18-hACE2 mice drives a rapid progression of the disease, including massive brain infection, which is more severe than the neuroinvasion reported in humans (Winkler et al., 2020; Song et al., 2021; Douaud et al., 2022). Some groups, pursuing more physiological models, mutated SARS-CoV-2S protein to use mouse ACE2 as receptor (Gu et al., 2020; Leist et al., 2020; Sun et al., 2020). Alternatively, WT mice susceptibility to new SARS-CoV-2 variants, like B.1.351/Beta or B.1.1.529/Omicron, could provide an opportunity to develop a model that better mimics the disease course, but introducing a new animal reservoir for the virus.

There is growing evidence that SARS-CoV-2 variants can induce diverse disease patterns in K18-hACE2 mice (Radvak et al., 2021). The objective of this work was to compare the infectivity of B.1, B.1.351/Beta, B.1.617.2/Delta and BA.1.1/Omicron SARS-CoV-2 variants in both hACE2 transgenic and WT mouse models and to characterize the progression of infection and pathological outcomes. B.1.351/Beta displayed a faster disease progression in hACE2 transgenic mice, while expanding tropism to WT mice. Strikingly, BA.1.1/Omicron was capable of infecting both transgenic and WT mice, and in both models without relevant clinical signs. These results were consistent with molecular modelling data and may have implications for SARS-CoV-2 ecology.

## Materials and Methods

### Biosafety Approval and Virus Isolation

The biologic biosafety committee of Germans Trias i Pujol Research Institute (IGTP) approved the execution of SARS-CoV-2 experiments at the BSL3 laboratory of the Centre for Bioimaging and Comparative Medicine (CSB-20-015-M8; CMCiB, Badalona, Spain). The SARS-CoV-2 variants used in this study were isolated from nasopharyngeal swabs of hospitalized patients in Spain as described elsewhere (Perez-Zsolt et al., 2021; Rodon et al., 2021). Briefly, viruses were propagated in Vero E6 cells (CRL-1586; ATCC, Virginia, VA, United States) for two passages and recovered by supernatant collection. The sequences of the four SARS-CoV-2 variants tested here were deposited at the GISAID Repository^1^ with accession IDs EPI_ISL_510689, EPI_ISL_1663571, EPI_ISL_3342900 and EPI_ISL_8151031 (Supplementary Figure 1). EPI_ISL_510689 was the first SARS-CoV-2 virus isolated in Catalonia in March 2020 and, compared to the Wuhan/Hu-1/2019 (WH1) strain, this isolate had the S protein mutations D614G, which is associated with the B.1 lineage, and R682L. EPI_ISL_1663571 was isolated in February 2021 and showed the mutations associated with the B.1.351/Beta variant (Supplementary Figure 1). EPI_ISL_3342900 was isolated in May 2021 and displayed the typical mutations associated with B.1.617.2/Delta (Supplementary Figure 1). Finally, EPI_ISL_8151031 was isolated in December 2021 and belonged to the Omicron sublinage B.1.1.529.1.1 (BA.1.1). Viral stocks were titrated to use equivalent TCID_50_/mL on Vero E6 cells using the Reed-Muench method and sequential 1/10 dilutions of the viral stocks as described previously (Rodon et al., 2021).

### Animal Procedures and Study Design

All animal procedures were performed under the approval of the Committee on the Ethics of Animal Experimentation of the IGTP and the authorization of *Generalitat de Catalunya* (code: 11222).

B6.Cg-Tg(K18-ACE2)2Prlmn/J (or K18-hACE2) hemizygous transgenic mice (034860, Jackson Immunoresearch, West Grove, PA, United States) were bred at CMCiB by pairing hemizygous males for Tg(K18-ACE2)2Prlmn (or K18-hACE2) with non-carrier B6.Cg females. The genotype of the offspring regarding Tg(K18-ACE2)2Prlmn was determined by qPCR at the IGTP’s Genomics Platform. Animals were kept in the BSL3 facility during the whole experiment.

A total of 99 adult mice (aged 3–6 months) were used in this experiment (55 K18-hACE2 hemizygous mice and 44 non-transgenic WT mice from the same breed). All groups were sex balanced. Infections were performed in different experiments between November 2020 and January 2022. Mice were anaesthetized with isoflurane (FDG9623; Baxter, Deerfield, IL, USA) and infected with B.1 (16 K18-hACE2 and 10 WT), B.1.351/Beta (12 K18-hACE2 and 10 WT), B.1.617.2/Delta (5 K18-hACE2 and 10 WT), or BA.1.1/Omicron (10 K18-hACE2 and 10 WT) SARS-CoV-2 isolates, or PBS (uninfected control group: 12 K18-hACE2 and 4 WT). Infection was performed using 1,000 TCID_50_ in 50 μl of PBS (25 μl/nostril). All mice fully recovered from the infection and anesthesia procedures.

After SARS-CoV-2 infection, body weight and clinical signs were monitored daily. Five WT animals per group were euthanized 3 days post-infection (dpi) for viral RNA quantification and pathological analyses. All remaining animals were euthanized 7 dpi or earlier if they reached a humane endpoint (body weight loss superior to 20% of the initial body weight and/or the display of moderate to severe neurological signs resulting from brain infection). Euthanasia was performed under deep isoflurane anesthesia by whole blood extraction *via* cardiac puncture and was confirmed by cervical dislocation. Serum was recovered from whole blood following 10 min centrifugation at 4,000 × *g*. Oropharyngeal swab, lung, brain, and nasal turbinate were collected for viral RNA quantification. Also, the three mentioned tissues were taken and fixed by immersion in 10% buffered formalin for histological and immunohistochemistry analyses.

### SARS-CoV-2 PCR Detection and Viral Load Quantification

Viral RNA was quantified in several samples (oropharyngeal swab, lung, brain, and nasal turbinate). Briefly, a piece of each tissue (100 mg approximately) was collected in 1.5 ml Sarstedt tubes (72,607; Sarstedt, Nümbrecth, Germany) containing 500 μl of DMEM medium (11,995,065; ThermoFisher Scientific) supplemented with 1% penicillin–streptomycin (10,378,016; ThermoFisher Scientific, Waltham, MA, United States). A 1.5 mm Tungsten bead (69,997; QIAGEN, Hilden, Germany) was added to each tube and samples were homogenized twice at 25 Hz for 30 s using a TissueLyser II (85,300; QIAGEN) and centrifuged for 2 min at 2,000 × *g*. Supernatants were stored at −80°C until use. RNA extraction was performed by using Viral RNA/Pathogen Nucleic Acid Isolation kit (A42352, ThermoFisher Scientific), optimized for a KingFisher instrument (5,400,610; ThermoFisher Scientific), following the manufacturer’s instructions. PCR amplification was based on the 2019-Novel Coronavirus Real-Time RT-PCR Diagnostic Panel guidelines and protocol developed by the American Center for Disease Control and Prevention (CDC-006-00019, v.07). Briefly, a 20 μl PCR reaction was set up containing 5 μl of RNA, 1.5 μl of N2 primers and probe (2019-nCov CDC EUA Kit, cat num 10,006,770, Integrated DNA Technologies, Coralville, IA, USA) and 10 μl of GoTaq 1-Step RT-qPCR (Promega, Madison, WI, USA). Thermal cycling was performed at 50°C for 15 min for reverse transcription, followed by 95°C for 2 min and then 45 cycles of 95°C for 10 s, 56°C for 15 s and 72°C for 30 s in the Applied Biosystems 7,500 or QuantStudio5 Real-Time PCR instruments (ThermoFisher Scientific). For absolute quantification, a standard curve was built using 1/5 serial dilutions of a SARS-CoV2 plasmid (2019-nCoV_N_Positive Control, catalog number 10006625, 200 copies/μL, Integrated DNA Technologies) and run in parallel in all PCR determinations. Viral RNA of each sample was quantified in triplicate and the mean viral RNA (in copies/mL) was extrapolated from the standard curve and corrected by the corresponding dilution factor. Mouse *gapdh* gene expression was measured in duplicate for each sample using TaqMan^®^ gene expression assay (Mm99999915_g1; ThermoFisher Scientific) as amplification control.

### Histopathological and Immunohistochemical Analyses

Formalin-fixed lung, nasal turbinate, and brain samples were routinely processed for histopathology, and haematoxylin & eosin-stained slides were examined under optical microscope in a blinded fashion. A semi-quantitative approach based on the amount of inflammation (none, mild, moderate, or severe) was used to establish the damage caused by SARS-CoV-2 infection in mice, following a previously published scoring system (Brustolin et al., 2021; Vidal et al., 2021).

An immunohistochemistry (IHC) technique to detect SARS-CoV-2 nucleoprotein (NP) antigen using the rabbit monoclonal antibody (40143-R019, Sino Biological, Beijing, China) at a 1:15,000 dilution, was applied on nasal turbinate, lung, and brain sections from all animals. The amount of viral antigen in tissues was semi-quantitatively scored in a blinded fashion (low, moderate, and high amount, or lack of antigen detection; Brustolin et al., 2021; Vidal et al., 2021).

### Quantification of Humoral Responses Against SARS-CoV-2

The humoral response against SARS-CoV-2 was evaluated using an in-house ELISA, as previously reported (Brustolin et al., 2021). All samples were tested in the same plate in antigen coated and antigen-free wells for background subtraction. Briefly, half Nunc Maxisorp ELISA plates were coated with 50 μl of S protein (SinoBiologicals, Beijing, China), all at 1 μg/ml in PBS and incubated overnight at 4°C. Then, plates were washed and blocked using PBS/1% of bovine serum albumin (BSA, Miltenyi biotech) for 2 h at room temperature. After that, duplicates of each sample were added in the same plate to the wells coated with and without antigen. Samples were assayed at 1/200 dilution in blocking buffer overnight at 4°C. The next day, after washing, plates were incubated for 1 h at room temperature with HRP conjugated- (Fab)2 Goat anti-mouse IgG (Fc specific) or Goat anti-mouse IgM (both 1/10000, Jackson Immunoresearch). Then, plates were washed and revealed with o-Phenylenediamine dihydrochloride (OPD, Sigma Aldrich) and stopped using 2 N of H_2_SO_4_ (Sigma Aldrich). The signal was analyzed as the optical density (OD) at 492 nm with noise correction at 620 nm. The specific signal for each antigen was calculated as corrected optical density after subtracting the background signal obtained for each sample in antigen-free wells.

### Viral Titration of Replicative SARS-CoV-2 in the Lungs of WT Mice

Lung tissues sampled at 3 or 7 dpi were evaluated for the presence of replicative virus by titration in Vero E6 cells as previously described (Haagmans et al., 2016; Rodon et al., 2021). Briefly, after tissue homogenization, each sample was sequentially diluted in 10-fold increments in duplicate, transferred in a 96 well plate on a Vero E6 cells monolayer and incubated at 37°C and 5% CO_2_. Plates were monitored daily under the microscope, and, at 5 dpi, wells were evaluated for the presence of cytopathic effects. The amount of infectious virus was calculated by determining the TCID_50_ using the Reed–Muench method.

### Molecular Modelling of ACE2 Species With SARS-CoV-2 Spike

Since no experimental structure of murine ACE2 (mACE2) in complex with the S protein of SARS-CoV-2 was available, we ran MODELLER v10.1 (Šali and Blundell, 1993) to generate homology models. As a template, the crystallographic structure of hACE2 in complex with the S RBD of the initial virus variant (PDB id 6M0J), which is the same as the RBD of the B.1 variant, was used. We created a total number of 10 models (identified as mACE2-B.1 RBD). In a second step, using the MODELLER models as input, we ran FoldX v5 (Schymkowitz et al., 2005; Delgado et al., 2019) to model the mutations at the RBD associated with the three VOCs under study: B.1.351/Beta, B.1.617.2/Delta and BA.1/Omicron (Supplementary Figure 1). We obtained 10 additional models for each variant labeled mACE2-B.1.351/Beta RBD, mACE2-B.1.617.2/Delta RBD and mACE2-BA.1/Omicron (Supplementary Figure 2). FoldX parameters had default values except vdwDesign that was set to zero. To estimate the binding affinity changes induced by the mutations of the VOCs, we evaluated each model with FoldX. As a control, we repeated the procedure described above to generate models of hACE2 in complex with the RBD of the B.1 virus (hACE2 – B.1 RBD), the B.1.351/Beta variant (hACE2 – B.1.351/Beta RBD), the B.1.617.2/Delta variant (hACE2 – B.1.617.2/Delta RBD), and the BA.1/Omicron variant (hACE2 – BA.1/Omicron RBD). As before, we ended up with 10 different structural models per complex type. We visualized and produced the pictures of the models with UCSF Chimera (Pettersen et al., 2004).

### Statistical Analyses

All figures were generated in GraphPad Prism 9.0.0. Statistical analyses were performed using R v4.1.1. Unpaired datasets were analyzed using a Kruskal-Wallis with Connover’s pairwise *post hoc* tests and adjusted for multiple testing using Holm’s method. Histopathological and IHC scores were compared using an Independence Asymptotic Generalized Pearson Chi-Squared Test for ordinal data. Datasets with abundance of data below the limit of detection were analyzed using a Petro-Prentice generalized Wilcoxon test with correction for multiple comparisons. For unpaired pairwise comparisons, a Mann–Whitney test was employed.

## Results

### SARS-CoV-2 Variants Induce Heterogeneous Disease Outcomes in K18-hACE2 Transgenic Mice

K18-hACE2 transgenic mice were experimentally infected intranasally with SARS-CoV-2 B.1 (*n* = 16), B.1.351/Beta (*n* = 10), B.1.617.2/Delta (*n* = 5) or BA.1.1/Omicron (*n* = 10) variants. Uninfected control K18-hACE2 animals received intranasal injection of PBS (*n* = 14).

**Figure 1:**
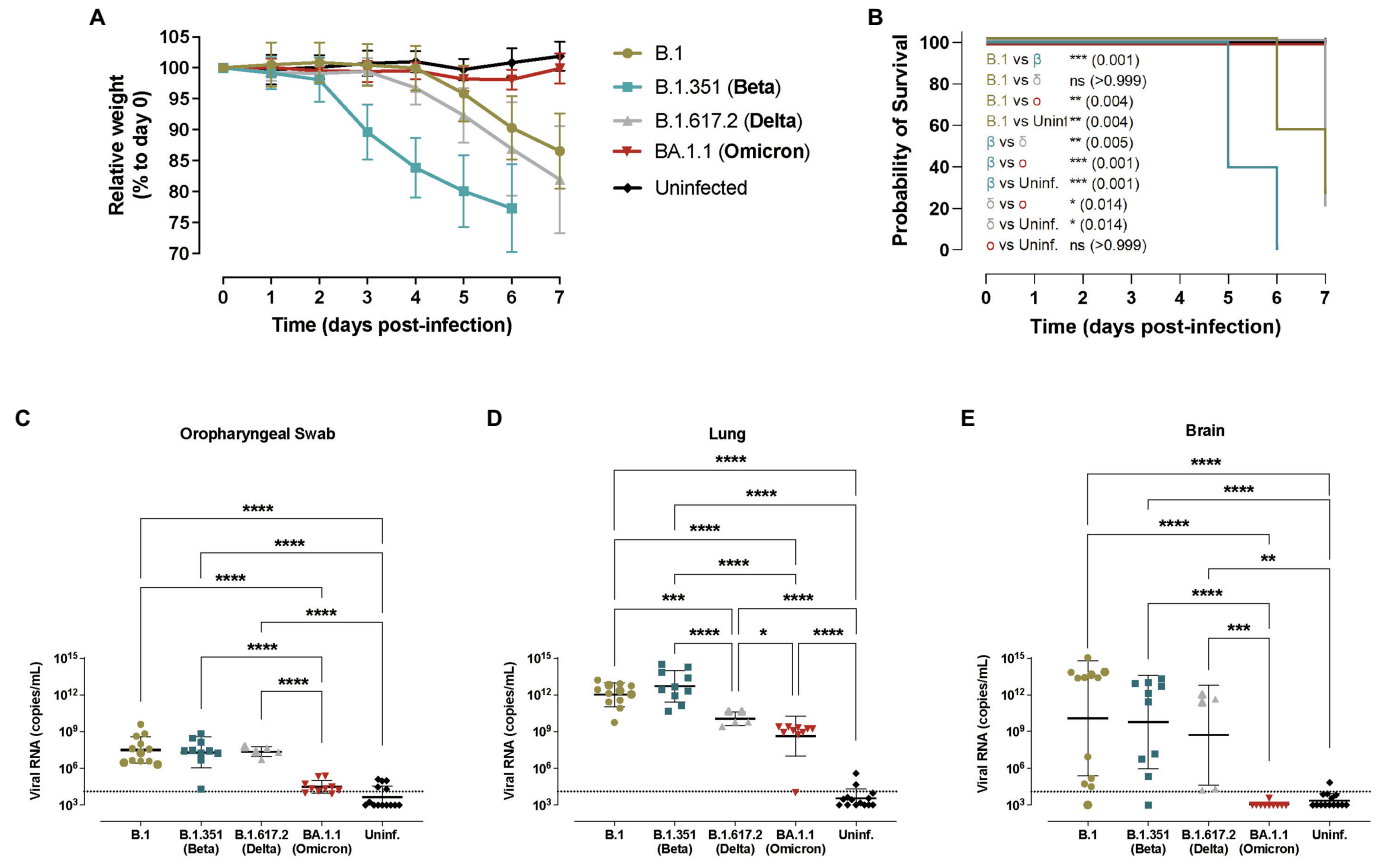
Progression of SARS-CoV-2 infection in hACE2+ mice. Transgenic K18-hACE2 mice were inoculated with SARS-CoV-2 variants: B.1 (gold, *n* = 12), B.1.351/Beta (blue, *n* = 10), B.1.617.2/Delta (grey, *n* = 5) and BA.1.1/Omicron (red, *n* = 10) or uninfected (black, *n* = 12). **(A)** Relative body weight follow-up of K18-hACE2 transgenic mice referred to day 0. Solid lines show the mean ± SD. Statistical differences at each timepoint were identified using a Kruskal-Wallis with Connover’s all-pairs test multiple comparisons (^*^p < 0.05; ^**^p < 0.01; ^***^p < 0.001; ^****^p < 0.0001; Supplementary Table 1). **(B)** Survival curve of K18-hACE2 transgenic mice. Statistical differences were identified using a Mantel-Cox test with Bonferroni’s correction for multiple comparisons (^*^p < 0.05; ^**^p < 0.01; ^***^p < 0.001). c-e) SARS-CoV-2 viral RNA load (copies/mL) on different tissues at endpoint: **(C)** oropharyngeal swab; **(D)** lung; **(E)** brain. Dashed line represents the limit of quantification. Statistical differences were identified using a Kruskal-Wallis with Connover’s all-pairs test multiple comparisons (^*^p < 0.05; ^***^p < 0.001; ^****^p < 0.0001).

Animal weight was monitored daily and B.1.351/Beta-infected transgenic mice started losing weight as early as 3 dpi (Figure 1A), which was significantly faster compared to the other variants (Supplementary Table 1). B.1- and B.1.617.2/Delta-infected animals started losing weight at 5 dpi. No major weight variation was observed in BA.1.1/Omicron-infected mice (Figure 1A).

Considering the survival rate, 2 out of 16 B.1-infected transgenic mice met humane endpoint criteria by 6 dpi (*n* = 7) and 7 dpi (*n* = 5), while four out of five B.1.617.2/Delta-infected transgenic animals reached humane endpoint at 7 dpi (Figure 1B). In comparison, all animals infected with the B.1.351/Beta variant met humane endpoint criteria by 5–6 dpi (6 at 5 dpi and 4 at 6 dpi), confirming a significant acceleration of the disease progression (*p* < 0.01, Figure 1B). Notably, none of the BA.1.1/Omicron-infected mice reached humane endpoint.

Virological analysis in oropharyngeal swabs, lung, brain, and nasal turbinate revealed widespread infection in B.1-, B.1.351/Beta- and B.1.617.2/Delta-infected K18-hACE2 transgenic mice (Figures 1C–E and Supplementary Figure 3A). Significant differences in viral RNA at endpoint between these three variants were only detected in the lungs, where the concentration was lower in animals infected with the B.1.617.2/Delta VOC, while B.1.351/Beta-infected mice showed a trend towards higher amounts of RNA (*p* = 0.1264; Figure 1D). However, at least for the B.1.351/Beta variant, these observed differences could be explained by the earlier euthanasia (5–6 dpi). SARS-CoV-2 viral RNA in the brain was very heterogeneous with all 3 variants (Figure 1E). Finally, animals infected with the BA.1.1/Omicron variant consistently had lower viral RNA in all the tissues, and no viral RNA was detected in the brain (Figure 1E).

**Figure 2:**
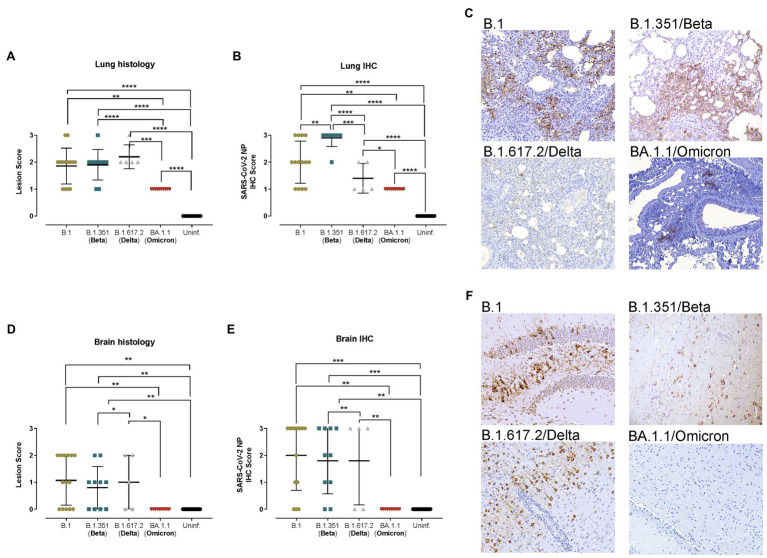
Histopathological and immunohistochemical analyses of lungs and brains from hACE2+ mice. Transgenic K18-hACE2 mice were inoculated with SARS-CoV-2 variants: B.1 (gold, *n* = 16), B.1.351/Beta (blue, *n* = 10), B.1.617.2/Delta (grey, *n* = 5) and BA.1.1/Omicron (red, *n* = 10) or uninfected (black, *n* = 16). **(A)** Lung histopathological scoring of broncho-interstitial pneumonia in K18-hACE2 transgenic mice at endpoint. **(B)** SARS-CoV-2 IHC scoring in lungs of K18-hACE2 transgenic mice at endpoint. **(C)** Lung IHC from a B.1-, B.1.351/Beta-, B.1.617.2/Delta-, and BA.1.1/Omicron-infected K18-hACE2 transgenic mice at 7 dpi, 5 dpi, 7 dpi and 7 dpi, respectively. **(D)** Brain histopathological scoring of non-suppurative meningoencephalitis in K18-hACE2 transgenic mice at endpoint. **(E)** SARS-CoV-2 NP IHC scoring in the brain of K18-hACE2 transgenic mice at endpoint. **(F)** Brain IHC from a B.1-, B.1.315/Beta-, B.1.617.2/Delta-, BA.1.1/Omicron-infected K18-hACE2 transgenic mice at 7 dpi, 5 dpi, 7 dpi and 7 dpi, respectively. All immunohistochemical slides were Hematoxylin counterstained. Lesion scoring: 0 – no lesion, 1 – mild lesion, 2 – moderate lesion, 3 – severe lesion. IHC scoring: 0 – no antigen, 1 – low and multifocal antigen, 2 – moderate and multifocal antigen, 3 – high and diffuse antigen. Statistical differences were identified using an Independence Asymptotic Generalized Pearson Chi-Squared Test for ordinal data (^*^p < 0.05; ^**^p < 0.01; ^***^p < 0.001; ^****^p < 0.0001).

The histopathological analysis of the lungs showed similar lesions in B.1-, B.1.351/Beta-, and B.1.617.2/Delta-infected animals, which displayed mild to moderate broncho-interstitial pneumonia at endpoint, and in some cases achieved severe pneumonia (Figure 2A). Furthermore, SARS-CoV-2 NP detection in the lungs by IHC was consistent with virological data, since animals infected with the B.1.351/Beta variant had a higher score and those infected with the B.1.617.2/Delta VOC had a lower score than B.1 (Figures 2B,C). These differences, however, could be associated with a higher replication or an earlier euthanasia in the B.1.351/Beta-infected group. Animals infected with the BA.1.1/Omicron variant clearly showed lower lesion and IHC scores in the lungs (Figure 2C).

In line with the viral RNA results, the brains of K18-hACE2 transgenic mice displayed a high heterogeneity of both lesions (multifocal non-suppurative meningo-encephalitis; Figure 2D) and amount of viral antigen (Figure 2E). Some animals had mild to moderate lesions and displayed low to very high antigen levels (Figure 2F), while others had no lesions nor antigen in the analyzed areas. These results were consistent within the different SARS-CoV-2 variants, except for BA.1.1/Omicron, in which neither lesions nor virus were detected (Figure 2F).

A sex-bias analysis was performed for all the parameters analyzed. No significant differences were found between females and males considering weight loss, survival, viral RNA, and IHC and histological scores (Supplementary Figure 3) in any of animal groups.

Finally, nasal turbinate analysis in transgenic mice showed no evident inflammatory lesions upon infection with SARS-CoV-2 variants (data not shown), and only low antigen amounts (Supplementary Figures 4B–D).

### B.1.351/Beta and BA.1.1/Omicron VOCs Can Infect WT Mice Promoting a Mild-Like Disease

**Figure 3:**
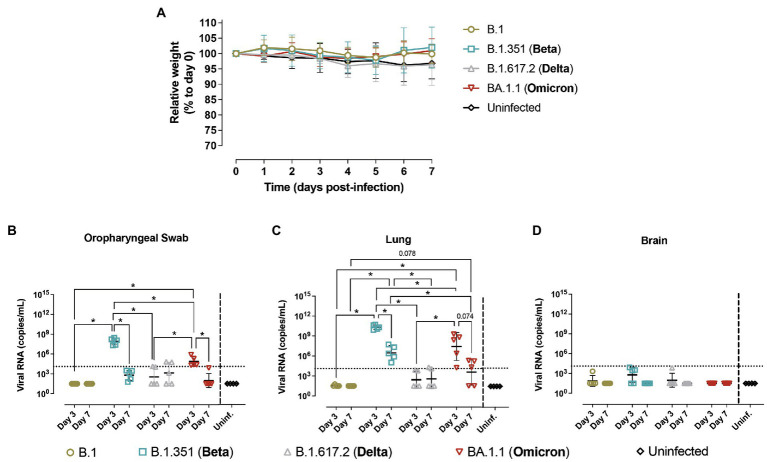
Progression of SARS-CoV-2 infection in hACE2- mice. Wildtype (WT) mice were inoculated with SARS-CoV-2 variants: B.1 (gold, *n* = 10), B.1.351/Beta (blue, *n* = 10), B.1.617.2/Delta (grey, *n* = 10) and BA.1.1/Omicron (red, *n* = 10) or uninfected (black, *n* = 4). Animals were euthanized at 3 dpi (*n* = 5) and 7 dpi (*n* = 5). **(A)** Relative body weight follow-up of WT mice referred to 0 dpi. Solid lines show the mean ± SD. b-d) SARS-CoV-2 viral RNA load (copies/mL) on different tissues: **(B)** oropharyngeal swab; **(C)** lung; **(D)** brain. Dashed line represents the limit of quantification. For statistical analysis, all data below the limit of detection (2.04) were censored. Statistically significant differences were identified using a Petro-Prentice generalized Wilcoxon test with correction for multiple comparisons (^*^p < 0.05).

In parallel, hACE2- negative mice (WT mice) from the same breed were infected with B.1 (*n* = 10), B.1.351/Beta (*n* = 10), B.1.617.2/Delta (*n* = 10), or BA.1.1/Omicron (*n* = 10) variants. Control included 4 uninfected animals. Five animals per infected group were euthanized at 3 dpi and the other five at 7 dpi, together with the uninfected animals. WT animals did not display significant body weight alterations with any of the variants tested (Figure 3A).

Virological analysis in oropharyngeal swabs, lung and nasal turbinate showed the presence of viral RNA in WT animals infected with both B.1.351/Beta and BA.1.1/Omicron (Figures 3B,C and Supplementary Figure 4). Notably, viral RNA at 3 dpi was higher than that at 7 dpi. No viral genetic material was detected in the brain (Figure 3D). Viral RNA levels in WT mice infected with B.1 and B.1.617.2/Delta were very low or undetectable, and overall showing no significant differences with uninfected animals (Figures 3B–D).

**Figure 4:**
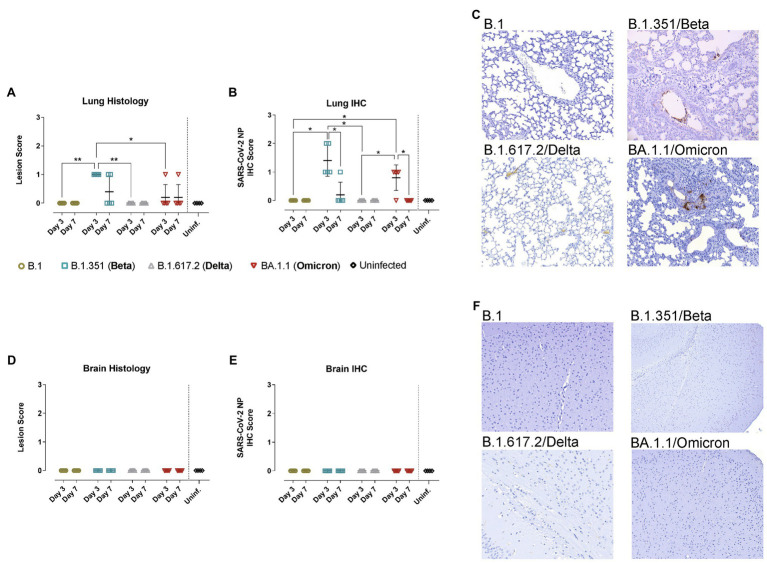
Histopathological and immunohistochemical analyses of lungs and brains from hACE2- mice. WT mice were inoculated with SARS-CoV-2 variants: B.1 (gold, *n* = 10), B.1.351/Beta (blue, *n* = 10), B.1.617.2/Delta (grey, *n* = 10), BA.1.1/Omicron (red, *n* = 10) or uninfected (black, *n* = 4). Animals were euthanized at 3 dpi (*n* = 5) and 7 dpi (*n* = 5). **(A)** Lung histopathological scoring of broncho-interstitial pneumonia in WT mice. **(B)** SARS-CoV-2 IHC scoring in lungs of WT mice. **(C)** Lung IHC from a B.1-, B.1.315/Beta-, B.1.617.2-, and BA.1.1/Omicron-infected WT mouse at 3 dpi. **(D)** Brain histopathological scoring of non-suppurative meningoencephalitis in WT mice. **(E)** SARS-CoV-2 IHC scoring in the brain of WT mice. **(F)** Brain IHC from a B.1-, B.1.315/Beta-, B.1.617.2-, and BA.1.1/Omicron-infected WT mouse at 3 dpi. All immunohistochemical slides were hematoxylin counterstained. Lesion scoring: 0 – no lesion, 1 – mild lesion, 2 – moderate lesion, 3 – severe lesion. IHC scoring: 0 – no antigen, 1 – low and multifocal antigen, 2 – moderate and multifocal antigen, 3 – high and diffuse antigen. Statistical differences were identified using an Independence Asymptotic Generalized Pearson Chi-Squared Test for ordinal data (^*^p < 0.05; ^**^p < 0.01).

Consistently, pathological assessment and SARS-CoV-2 detection by IHC in the lungs of WT mice infected with B.1.351/Beta and BA.1.1/Omicron, but not with B.1 nor B.1.617.2/Delta, demonstrated mild infection-related lesions and low amounts of viral antigens mainly at 3 dpi (Figures 4A–C). Additionally, neither lesions nor viral antigens were detected in the brain of WT mice infected with the different variants (Figures 4D–F).

**Figure 5:**
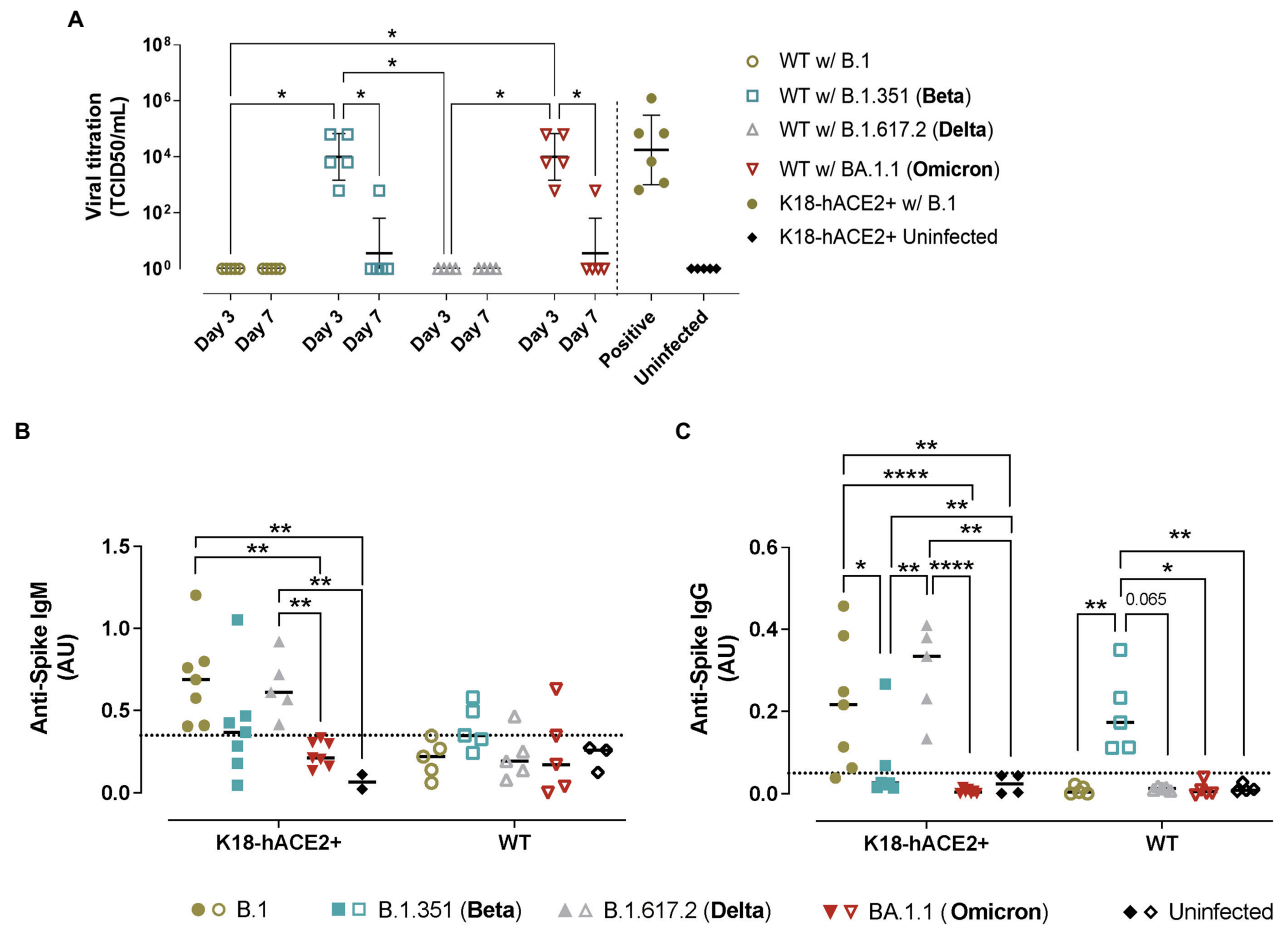
Viral titration and humoral immune responses in hACE2+ and hACE2- mice infected with different SARS-CoV-2 variants. K18-hACE2 transgenic mice (solid symbols) and WT mice (empty symbols) were infected with SARS-CoV-2 B.1 (gold), B.1.351/Beta (blue), B.1.617.2/Delta (grey) and BA.1.1/Omicron (red) variants. **(A)** Viral load of replicative virus (TCID_50_) recovered from lung samples from SARS-CoV-2-infected mice at endpoint in Vero E6 cells at day 5 of culture. For statistical analysis, all data below the limit of detection (Holmes et al., 2021) were censored. Statistically significant differences were identified using a Petro-Prentice generalized Wilcoxon test with correction for multiple comparisons (^*^p < 0.05). **(B)** Absorbance units of anti-S IgM from SARS-CoV-2-infected mice at endpoint. **(C)** Absorbance units of anti-S IgG. Dotted line represents the background calculated as the mean ± 2*SD of the absorbance units of uninfected mice both for IgM and IgG. Statistically significant differences were found using a Kruskal-Wallis with Connover’s all-pairs test multiple comparisons (^*^p < 0.05; ^**^p < 0.01; ^****^p < 0.0001).

Replicative SARS-CoV-2 was successfully recovered from the lungs of B.1.351/Beta and BA.1.1/Omicron but not B.1 nor B.1.617.2/Delta-infected WT animals (Figure 5A), fully demonstrating that the two former VOCs could productively infect WT mice. Consistent with all the previous data, SARS-CoV-2 viral load was higher at 3 dpi than 7 dpi, indicating that the virus was either cleared by the immune response or that it could not sustain long periods of infection.

### Humoral Responses Induced by SARS-CoV-2 Infection in Transgenic and WT Mice

In transgenic K18-hACE2 mice, the analysis of the humoral response against SARS-CoV-2 S protein (WH1) demonstrated a robust development of anti-S IgM and IgG in B.1 and B.1.617.2/Delta-infected mice at endpoint (Figures 5B,C). In contrast, the humoral response induced by B.1.351/Beta infection was detectable in some animals but not significant across groups. Importantly, B.1.351/Beta-infected animals were euthanized at earlier timepoints, which could explain this lower response. Animals infected with BA.1.1/Omicron did not display significant induction of antibodies against the WH1 S protein at 7 dpi.

In comparison, in WT mice, anti-S IgM and IgG antibodies were only observed in response to B.1.351/Beta infection (Figures 5B,C). On the one hand, the presence of anti-S antibodies after infection with B.1.351/Beta reinforced the idea that this variant could establish a productive infection. On the other hand, again, no clear humoral response against WH1 S protein was detected in BA.1.1/Omicron-infected WT mice, similar to K18-hACE2 transgenic mice.

### Molecular Modelling of Human and Murine ACE2 Interactions With SARS-CoV-2 Spike From VOCs

**Figure 6:**
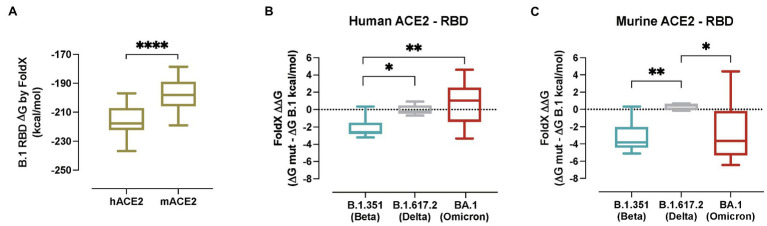
*In silico* modelling of the affinity of SARS-CoV-2 variants for human and murine ACE2. **(A)** Gibbs free energy of the FoldX models of hACE2 and mACE2 in complex with the S RBD from SARS-CoV-2 B.1 RBD. Complexes with lower energies are predicted as having higher binding affinities. Statistically significant differences were found using a Mann–Whitney test (^****^p < 0.0001). **(B)** Predicted binding affinity changes induced by VOCs B.1.351/Beta (blue), B.1.617.2/Delta (grey) and BA.1/Omicron (red) of the S RBD in complex with human ACE2. **(C)** Predicted binding affinity changes induced by VOCs B.1.351/Beta (blue), B.1.617.2/Delta (grey) and BA.1/Omicron (red) of the S RBD in complex with murine ACE2. Statistically significant differences were found using a Kruskal-Wallis with Connover’s all-pairs test multiple comparisons (^*^p < 0.05; ^**^p < 0.01; ^****^p < 0.0001).

To estimate the impact of the B.1.351/Beta, B.1.617.2/Delta and BA.1/Omicron associated RBD mutations on binding affinity, different models of the ACE2-S RBD complex were evaluated with FoldX. First, a model of B.1 RBD was built in complex with both the hACE2 and the mACE2, and as expected simulations yielded a higher affinity for the former (Figure 6A; *p* < 0.0001). Next, models of the RBDs containing the specific mutations of the B.1.351/Beta, B.1.617.2/Delta and BA.1/Omicron VOCs (Supplementary Figure 1) were evaluated, and the changes in binding affinity between B.1 and each of the variants were calculated (Figure 6B). Notably, the predicted changes in binding affinity of hACE2 in complex with the RBD of the different VOCs are in line with reported experimental values measured with surface plasmon resonance assay (SPR; Han et al., 2022). Computed energies were significantly lower for the B.1.351/Beta than for the B.1 variant complexes both in human and murine ACE2, suggesting that the mutations associated with B.1.351/Beta improve binding with ACE2 in both species (Supplementary Figure 5). In comparison, there were no significant differences in the binding affinity induced by the variant B.1.617.2/Delta neither in murine nor human ACE2. Finally, in the case of the BA.1/Omicron variant, computed energy was only lower for mACE2, indicating that the binding affinity of BA.1 RBD complexes increase for murine but not for human.

A detailed analysis of the B.1.351/Beta RBD complexes with human and murine ACE2 provided an insight in the effects of each mutation at the molecular level (Supplementary Figure 5). Mutation K417N caused the loss of a salt bridge between Lys417 and hACE2 Asp30, reducing the affinity to the human receptor. Interestingly, in mice, no salt bridge is formed between Lys417 and mACE2 Asn30, and binding affinity is not negatively affected by the mutation (Supplementary Figures 4A–D). A similar case occurred with mutation E484K, which induced the loss of a salt bridge between Glu484 and Lys31 in hACE2, but not in mACE2, where no salt bridge is formed between Glu484 and mACE2 Asn31 (Supplementary Figures 4E–H). Mutation N501Y, which is also present in BA.1/Omicron’s RBD, increased the number of hydrophobic interactions between Tyr501 and ACE2 Tyr41 both in human and mice. Additionally, Tyr501 can establish a cation-pi interaction with hACE2 Lys353 in human, and a pi-pi interaction with mACE2 His353 in mice (Supplementary Figures 4I–L). In summary, mutation N501Y should increase the affinity in both species. This effect would be partially reduced by the loss of salt bridges caused by mutations K417N and E484K in human, but not in mice.

## Discussion

WT mice are not susceptible to the ancestral SARS-CoV-2. Therefore, many strategies have been pursued to generate a mouse model capable of being infected by this virus, ranging from the use of transgenic and knock-in humanized mice (Gurumurthy et al., 2020) to the adaptation of the virus for the murine receptor (Leist et al., 2020).

K18-hACE2 transgenic mice are one of the most widely used animal models for the study of SARS-CoV-2, owing to its previous development and characterization during the SARS-CoV epidemics (Winkler et al., 2020). Here, we evaluated how this model behaves against B.1 and three SARS-CoV-2 VOCs (B.1.351/Beta, B.1.617.2/Delta and BA.1.1/Omicron). Among the multiple parameters analyzed in infected mice, we found significant differences in viral RNA concentration in the lungs at 7 dpi or humane endpoint. B.1.351/Beta-infected transgenic animals presented the highest viral burden and B.1.617.2/Delta displayed a lower amount of virus compared to B.1; however, we cannot rule out that the higher viral RNA associated to B.1.351/Beta is a consequence of an earlier euthanasia. In contrast, BA.1.1/Omicron-infected animals consistently presented the lowest amount of SARS-CoV-2 and lesions across all the tissues. The most relevant findings between variants in this model were that B.1.351/Beta-infected mice displayed a faster progression of the infection, while BA.1.1/Omicron did not manifest any detectable clinical sign. Interestingly, females and males infected with the same variant did not display any difference in all the analyzed parameters, consistent with previous data in K18-hACE2 mice (Winkler et al., 2020), while diverging from results observed in hamster models (Michita and Mysorekar, 2021). Overall, K18-hACE2 transgenic mice demonstrated to be a robust model for the study of SARS-CoV-2 infection, providing consistent readouts (viral RNA, IHC, histology) in animals infected with the same variant. However, major outcome differences were identified upon infection with B.1 or the three VOCs tested. B.1.351/Beta was the most pathogenic variant, B.1 and B.1.617.2/Delta variants showed an intermediate phenotype, and BA.1.1/Omicron was the least pathogenic VOC.

K18-hACE2 transgenic animals infected with B.1 and B.1.617.2/Delta elicited robust humoral responses (IgM and IgG), while antibody titers in animals infected with B.1.351/Beta were lower. This could likely result from the faster disease progression, which led to an earlier euthanasia, that interrupted the development of a complete humoral response. The lack of detection of anti-S antibodies in BA.1.1/Omicron-infected mice could be explained by the poor cross-reactivity of Omicron-elicited antibodies against the ancestral WH1 variant (Bartsch et al., 2021; Rössler et al., 2022). Alternatively, the milder infection induced by BA.1.1/Omicron might result in a lower elicitation of humoral responses, as reported in humans (Trinité et al., 2021b).

In comparison, virological, pathological, and serological analyses of WT mice infected with B.1 and SARS-CoV-2 VOCs unveiled how the B.1.351/Beta and BA.1.1/Omicron could establish productive infection, while B.1 and B.1.617.2/Delta could not, in accordance with previous findings (Kant et al., 2021; Montagutelli et al., 2021; Shuai et al., 2021; Zhang et al., 2022). Two relevant aspects of B.1.351/Beta and BA.1.1/Omicron SARS-CoV-2 infection in WT mice were, first, the lack of infection in the brain, and second, the transient viral replication in the lungs, detected at 3 dpi and subsequently cleared. Both observations could justify that WT mice, despite getting infected, did not develop severe neurological signs compared to transgenic hACE2 mice, since mACE2 is not as ubiquitously expressed in the brain of WT mice compared to hACE2 expression in K18-hACE2 mice (Qiao et al., 2020). Reduction of viral RNA in WT mouse tissues may reflect a rapid and effective viral clearance by the immune response, or the incapacity of the virus to sustain multiple cycles of replication.

Our molecular analyses predicted that B.1.351/Beta RBD mutations enhanced the affinity for both human and murine ACE2. This could help explain the enhanced infection progression in K18-hACE2 mice which express both ACE2 receptors. In comparison, the BA.1/Omicron variant displayed increased affinity only for the mACE2 receptor, suggesting that the reduced pathogenicity of this VOC is probably associated with other factors beyond RBD affinity for hACE2, such as lower fusogenicity (Meng et al., 2022; Suzuki et al., 2022). *In silico* predictions are also consistent with WT mice susceptibility to B.1.351/Beta and BA.1/Omicron infection. N501Y mutation, which is present both in B.1.351/Beta and BA.1/Omicron, may play a prominent role in that expanded species tropism. This is in line with previous findings showing *in vitro* how this mutation may contribute to an increased affinity for murine ACE2 (Thakur et al., 2021).

Overall, this study demonstrates that SARS-CoV-2 B.1.351/Beta and BA.1.1/Omicron display differential infectivity and pathology in K18-hACE2 mice. While B.1.351/Beta displays a faster progression than B.1 and B.1.617.2/Delta variants, the BA.1.1/Omicron variant courses as a milder infection with lower viral RNA at endpoint and no obvious clinical signs. However, both B.1.315/Beta and BA.1.1/Omicron VOCs can establish infection in WT mice, in line with previous findings (Kant et al., 2021; Montagutelli et al., 2021; Shuai et al., 2021; Zhang et al., 2022). Compared to hACE2-expressing transgenic mice that developed a severe clinical course and lesions upon SARS-CoV-2 infection, WT mice displayed a milder infection. In fact, both K18-hACE2 and WT mice infected with BA.1.1/Omicron achieved similar viral RNA amounts in the lungs, but WT mice had less lesions.

This milder outcome could be comparable to SARS-CoV-2 infection in other animal models, in which lung replication and viral pneumonia is detected, but eventually resolved by the action of the immune response (Kim et al., 2020). This, however, also raises concerns on how new variants, with increased affinity for ACE2 across multiple species, could expand their host tropism to animals that were initially refractory to SARS-CoV-2 infection, potentially generating new zoonotic reservoirs and increasing the risk of new spillover and spread of new variants in humans.

Further studies will prove whether, in a natural setting, increased affinity to mACE2 by B.1.351/Beta and BA.1.1/Omicron associated mutations, such as N501Y, can result in SARS-CoV-2 transmission between animals. Still, this study confirms the need to monitor SARS-CoV-2 VOCs beyond their interaction with humans (transmissibility, pathogenicity, and immune evasion).

## Data Availability Statement

The raw data supporting the conclusions of this article will be made available by the authors, without undue reservation.

## Ethics Statement

The animal study was reviewed and approved by Committee on the Ethics of Animal Experimentation of the Germans Trias i Pujol Institute and approved by “Generalitat de Catalunya” (code: 11222).

## Author’s Note

Unrelated to the submitted work, JC, JB, and BC are founders and shareholders of AlbaJuna Therapeutics, SL; BC is founder and shareholder of AELIX Therapeutics, SL; JB reports institutional grants from HIPRA and MSD; NI-U reports institutional grants from HIPRA, Pharma Mar, and Dentaid.

## Author Contributions

FT-F, BT, JC, and JB conceived and designed the experiments. FT-F, BT, and AP-G performed the animal procedures. FT-F, AP-G, MR-D, ER-M, EG-V, CÁ-N, and MP performed the analytical experiments. FT-F, BT, AP-G, MR-D, AV, VU, MN-J, JV-A, BC, EB, JC, JS, and JB analyzed and interpreted the data. DP-Z, JM-B, DR-R, NI-U, IB, CA, AA and TP provided key reagents. MR-D, VG, RL and AV performed the in silico analysis. FT-F, BT, AP-G, MR-D, JV-A, EB, JC, JS, and JB wrote the paper. All authors contributed to the article and approved the submitted version.

## Funding

The research of CBIG consortium (constituted by IRTA-CReSA, BSC & IrsiCaixa) is supported by Grifols. We thank Foundation Dormeur for financial support for the acquisition of the QuantStudio-5 real time PCR system. CÁ-N has a grant by Secretaria d’Universitats i Recerca de la Generalitat de Catalunya and Fons Social Europeu. EG-V is a research fellow from PERIS (SLT017/20/000090). This work was partially funded by grant PID2020-117145RB-I00 from the Spanish Ministry of Science and Innovation (NI-U) the Departament de Salut of the Generalitat de Catalunya (grant SLD016 to JB and Grant SLD015 to JC), the Spanish Health Institute Carlos III (Grant PI17/01518. PI20/00093 to JB and PI18/01332 to JC), Fundació La Marató de TV3 (Project202126-30-21), CERCA Programme/Generalitat de Catalunya 2017 SGR 252, and the crowdfunding initiatives #joemcorono (https://www.yomecorono.com), BonPreu/Esclat and Correos. Funded in part by Fundació Glòria Soler (JB). The funders had no role in study design, data collection and analysis, the decision to publish, or the preparation of the manuscript.

## Conflict of Interest

The authors declare that the research was conducted in the absence of any commercial or financial relationships that could be construed as a potential conflict of interest.

## Publisher’s Note

All claims expressed in this article are solely those of the authors and do not necessarily represent those of their affiliated organizations, or those of the publisher, the editors and the reviewers. Any product that may be evaluated in this article, or claim that may be made by its manufacturer, is not guaranteed or endorsed by the publisher.

## Footnotes

^1^ http://gisaid.org
