# Supplementary material for: Heterogeneous Infectivity and Pathogenesis of SARS-CoV-2 Variants Beta, Delta and Omicron in Transgenic K18-hACE2 and Wildtype Mice
Source: Front Microbiol. 2022 May 4;13:840757. doi: 10.3389/fmicb.2022.840757 (PMC9114491; doi:10.3389/fmicb.2022.840757)
Supplement: Supplementary file 1 [file Data_Sheet_1.PDF]

A

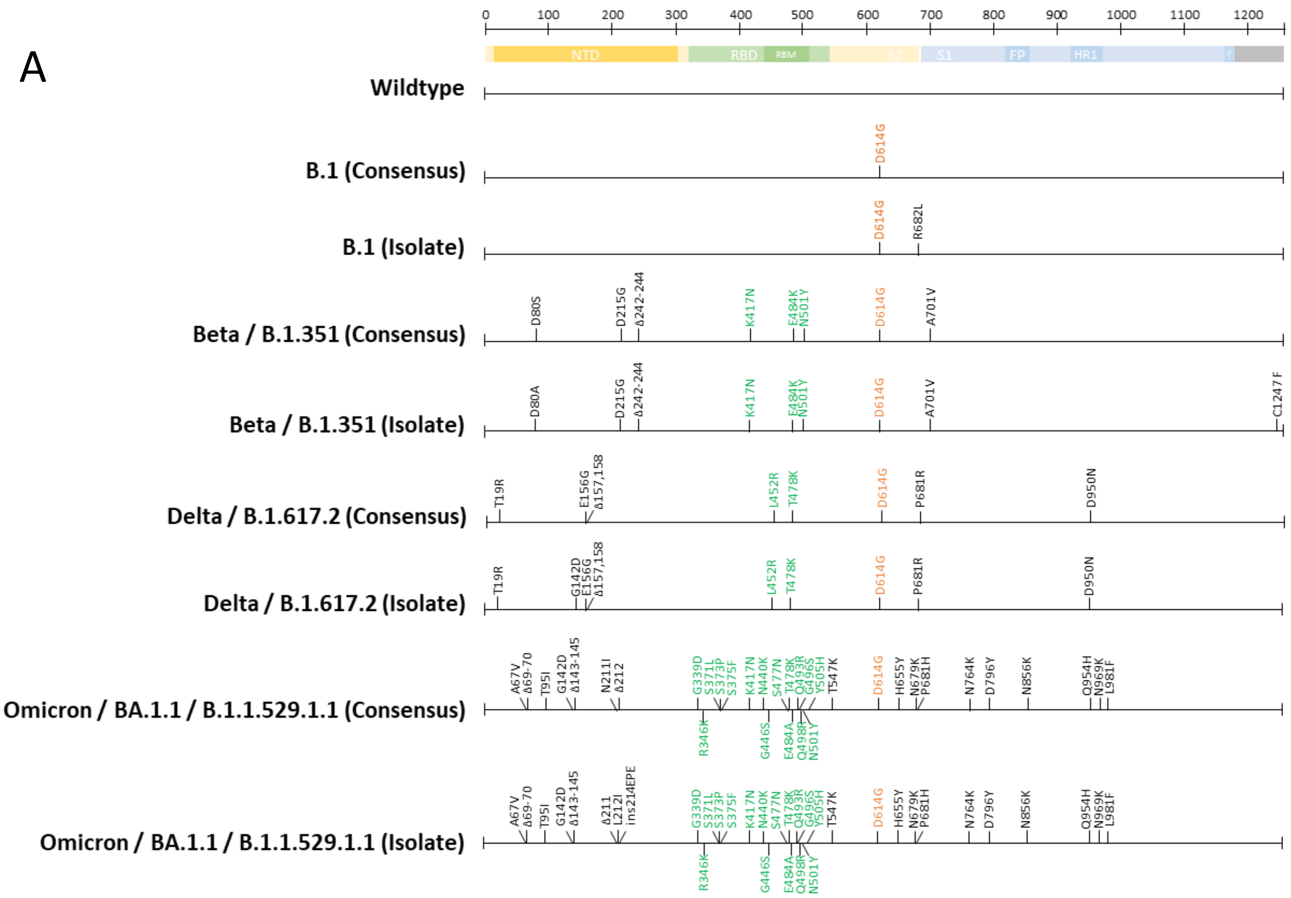

B

Genes	B.1 Isolate	B.1.351/Beta Isolate	B.1.617.2/Delta Isolate	BA.1.1/Omicron Isolate
N	-	P6L<br>T205I	D63G<br>**R203M**<br>G215C<br>D377Y	P13L<br>E31del<br>R32del<br>S33del<br>**R203K**<br>G204R
E	-	P71L	-	T9I
M	-	-	I82T	D3G<br>Q19E<br>A63T
NSPs	-	NSP2_T85I<br>NSP2_R544K<br>NSP3_S794L<br>NSP3_K837N<br>NSP5_K90R<br>**NSP6_S106del**<br>**NSP6_G107del**<br>NSP6_F108del<br>**NSP12_P323L**<br>NSP13_E261D	NSP3_A488S<br>NSP3_P1228L<br>NSP3_P1469S<br>NSP4_V167L<br>**NSP4_T492I**<br>NSP6_T77A<br>**NSP12_P323L**<br>NSP12_G671S<br>NSP13_P77L<br>NSP14_A394V	NSP3_K38R<br>NSP3_S1265del<br>NSP3_L1266I<br>NSP3_A1892T<br>**NSP4_T492I**<br>NSP5_P132H<br>NSP6_L105del<br>**NSP6_S106del**<br>**NSP6_G107del**<br>NSP6_I189V<br>**NSP12_P323L**<br>NSP14_I42V
Accessory	-	NS3_Q57H<br>NS3_S171L	NS3_S26L<br>NS7a_V82A<br>NS7a_T120I<br>NS7b_T40I	-

Supplementary Figure 1. **Mutations associated with the SARS-CoV-2 B.1, B.1.351/Beta, B.1.617.2/Delta and BA.1.1/Omicron variants and the mutations found in the isolates used in this experiment.** a) Schematic representation of the mutations found in the Spike protein referred to the wildtype or original strain. Each mutation is overlapped with the domain where it is located (S1: light yellow, RBD: light green, RBM: green, S2: light blue, FP & HR1: blue). b) Table defining the mutations found in the N, E, M, NSP and accessory proteins in the isolates used in this experiment.

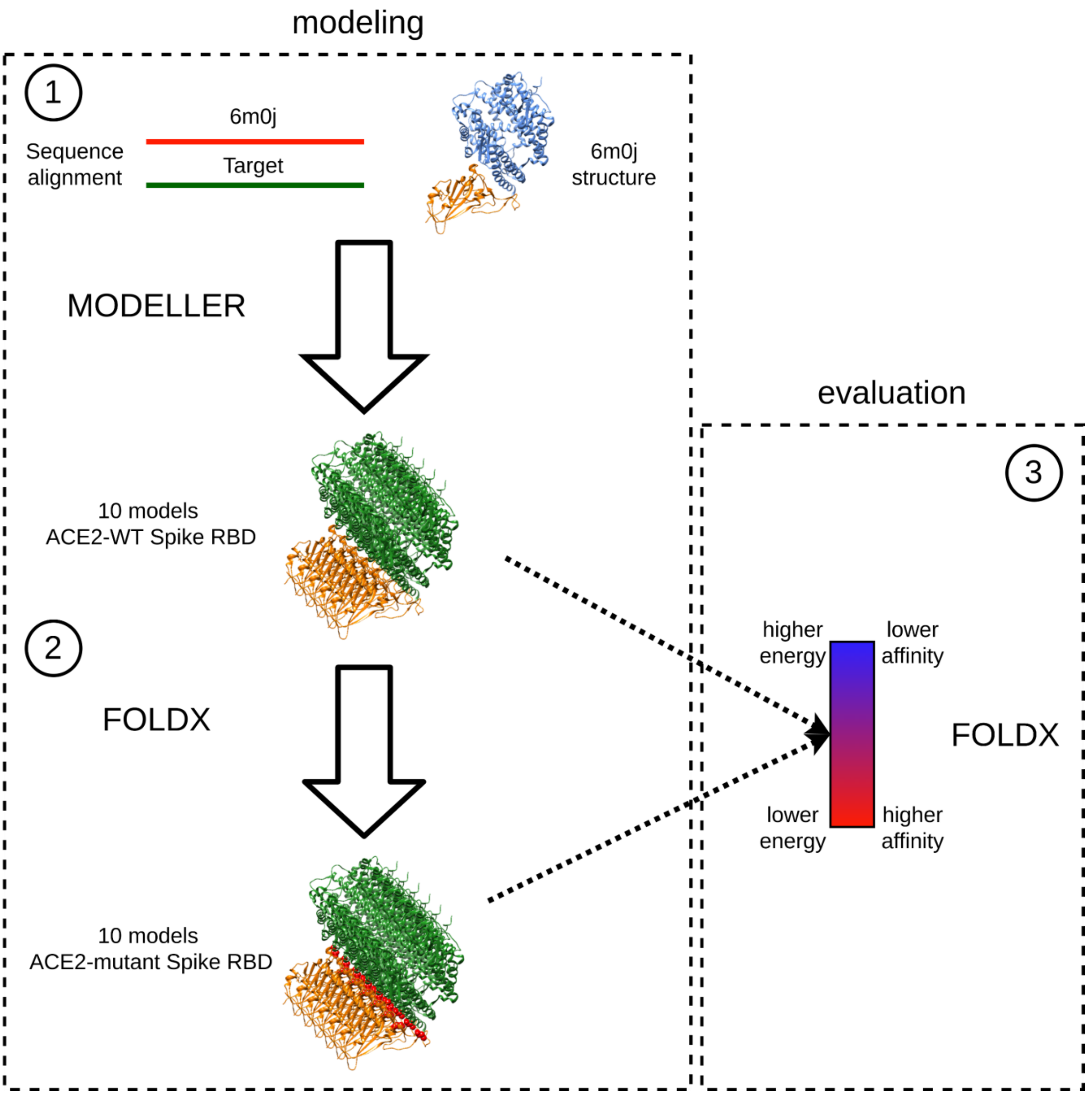

Supplementary Figure 2. **Protocol diagram for the *in silico* modelling of human and murine ACE2 in complex with SARS-CoV-2 B.1, B.1.351/Beta, B.1.617.2/Delta, and BA.1/Omicron variants**. 1) Ten models of ACE2 in complex with the spike RBD of the B.1 SARS-CoV-2 variant were created with MODELLER. The structure with PDB id 6M0J was used as a template. 2) Mutations associated with the B.1.351/Beta, B.1.617.2/Delta and BA.1.1/Omicron variants at the spike RBD were modelled with FoldX using MODELLER models as input. 3) All models were evaluated with FoldX to obtain an estimation of the binding affinity changes due to the mutations. The protocol was run twice: The first time to generate mACE2-RBD complexes, the second time to produce hACE2-RBD complexes to use as a control.

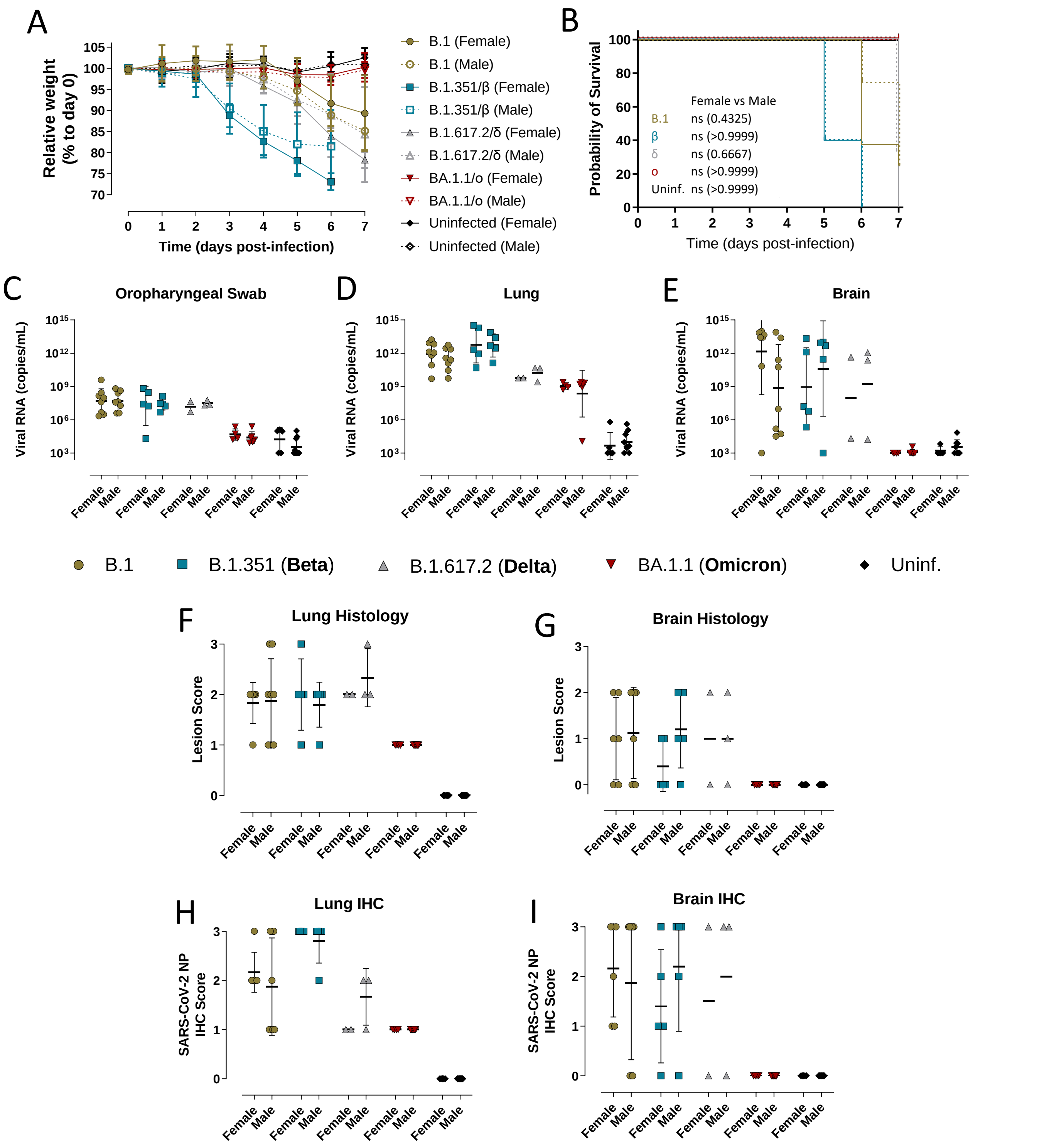

Supplementary Figure 3. **Sex-bias analysis of K18-hACE2 transgenic mice.** Transgenic K18-hACE2 mice were inoculated with SARS-CoV-2 variants: B.1 (gold, n=16), B.1.351/Beta (blue, n=10), B.1.617.2/Delta (grey, n=5) and BA.1.1/Omicron (red, n=10) or uninfected (black, n=12). a) Sex-bias analysis of the relative body weight of K18-hACE2 transgenic mice referred to day 0. Solid lines represent Females and dashed lines males. b) Sex-bias analysis of the survival curve of K18-hACE2 transgenic mice. Solid lines represent Females and dashed lines males. c-e) Sex-bias analysis of viral RNA loads from K18-hACE2+ transgenic mice challenged with SARS-CoV-2 VOCs in different tissues: c) oropharyngeal swab; d) lung; and e) brain. f-g) Sex-bias analysis of SARS-CoV-2 related lesions in K18-hACE2 mice challenged with different VOCs at different tissues: f) lung; and g) brain. f-g) Sex-bias analysis of SARS-CoV-2 detection by IHC against NP in K18-hACE2 mice challenged with different VOCs at different tissues: h) lung; and i) brain. No differences were found in any case (weight, survival, viral loads, histology and IHC) in any of the variants between females and males using a Kruskal-Wallis test with Dunn's comparison for multiple tests.

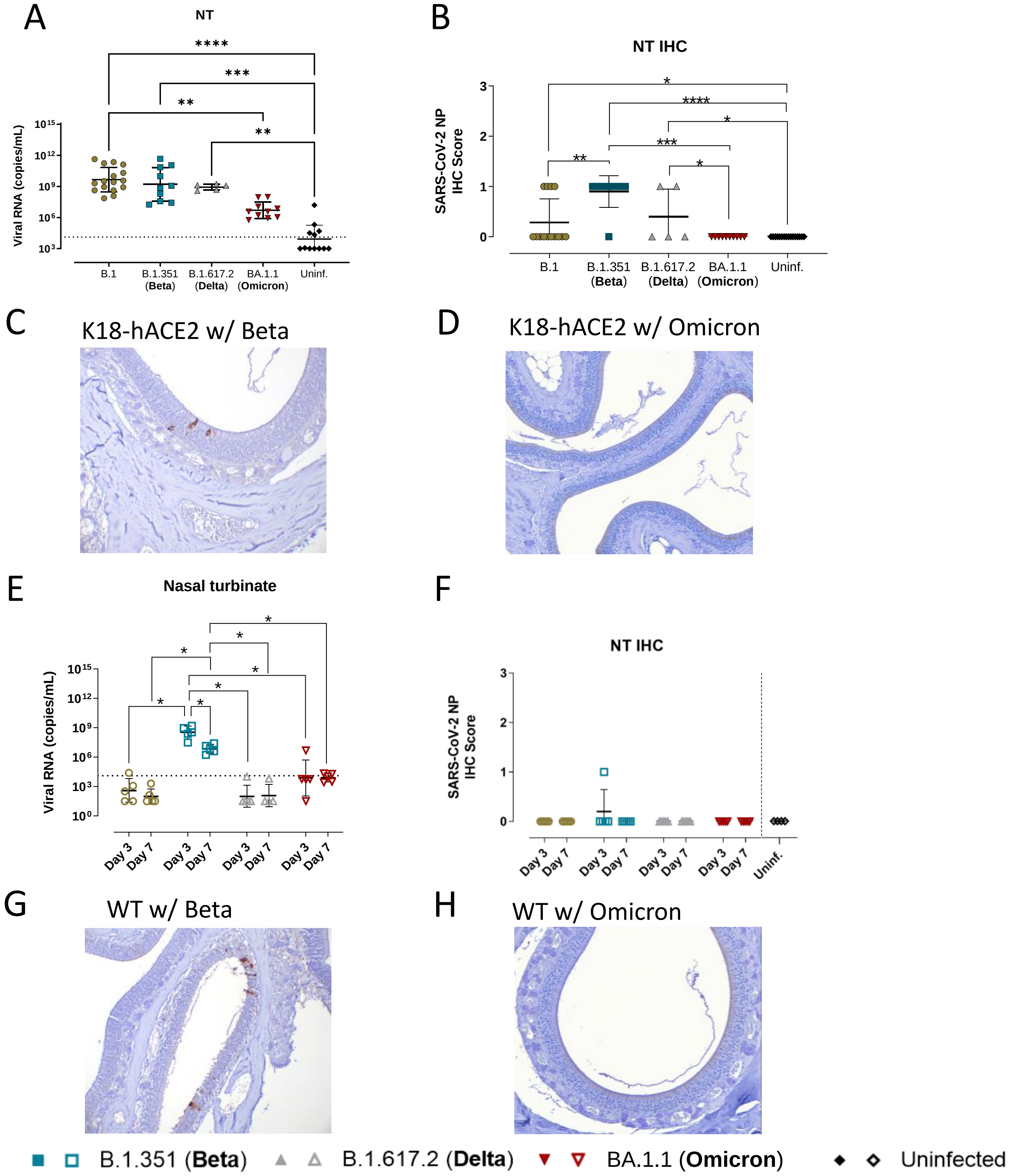

Supplementary Figure 4. **SARS-CoV-2 viral loads and lesions found in the nasal turbinate of hACE2+ and hACE2- mice**. a) Nasal turbinate viral RNA loads at endpoint in K18-hACE2 transgenic mice (solid symbols) challenged with SARS-CoV-2 B.1 (gold, n=16), B.1.351/Beta (blue, n=10), B.1.617.2/Delta (grey, n=5) and BA.1.1/Omicron (red, n=10) or uninfected (black, n=16). Statistical differences were identified using a Kruskal-Wallis with Connover's all-pairs test multiple comparisons (***p*<0.01; ****p*<0.001; *****p*<0.0001). b) SARS-CoV-2 NP protein IHC analysis in nasal turbinates' of K18-hACE2+ transgenic mice. Statistical differences were identified using an Independence Asymptotic Generalized Pearson Chi-Squared Test for ordinal data (*p<0.05; ***p*<0.01; ****p*<0.001; *****p*<0.0001). c) NT IHC from a B.1.351/Beta-infected K18-hACE2 mouse at 5 dpi. d) NT IHC from a B.1.1.529/Omicron-infected K18-hACE2 mouse at 7 dpi. e) Nasal turbinate viral RNA at endpoint in WT mice (empty symbols) challenged with SARS-CoV-2 B.1 (gold, n=10), B.1.351/Beta (blue, n=10), B.1.617.2/Delta (grey, n=10) and B.1.1.529/Omicron (red, n=10) or uninfected (black, n=4). Animals were euthanised at 3 dpi (n=5) and 7 dpi (n=5). f) SARS-CoV-2 NP protein IHC analysis in nasal turbinates of WT mice. g) NT IHC from a B.1.351/Beta-infected WT mouse at 3 dpi. h) NT IHC from a B.1.1.529/Omicron-infected WT mouse at 3 dpi.

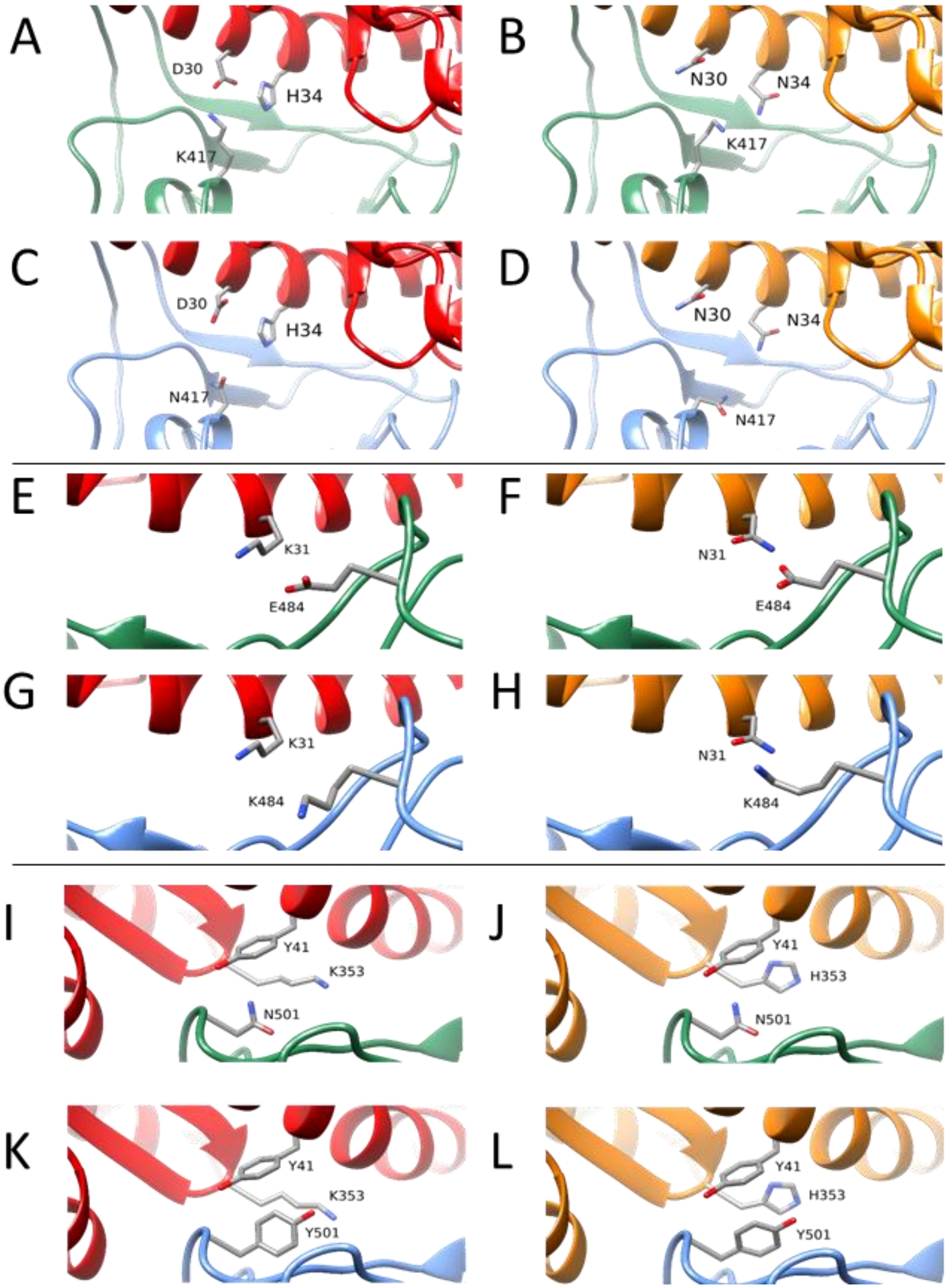

Supplementary Figure 5. **Structural details in models with hACE2 (red), mACE2 (orange), WT Spike (green) and B.1.351/Beta Spike (blue)**. In hACE2, mutation K417N causes the loss of a salt bridge between Lys417 and hACE2 Asp30, diminishing the binding affinity of the complex (a, c). In mice, no salt bridge is formed between WT RBD Lys417 and mACE2 Asn30, and affinity does not decrease by its loss (b, d). Similarly, mutation E484K induces the loss of a salt bridge between Glu484 and Lys31 in hACE2 (e, g), but not in mACE2, where no salt bridge is established between WT RBD Glu484 and mACE2 Asn31 (f, h). Mutation N501Y increases the number of hydrophobic interactions between Tyr501 and ACE2 Tyr41 in human and mice (i-l). Tyr501 can have a cation-pi interaction with hACE2 Lys353 in human (k), and a pi-pi interaction with mACE2 His353 in mice (l). The analysis suggests that the increase in affinity due to mutation N501Y is partially reduced by the loss of salt bridges in human, but not in mice.

Supplementary Table 1. Statistical comparison of weight loss between different VOCs.

	Day 0	Day 1	Day 2	Day 3	Day 4	Day 5	Day 6	Day 7
B.1 vs β	-	-	-	***	****	****	*	
B.1 vs δ	-	-	-	-	-	-	-	-
B.1 vs o	-	-	-	-	-	-	**	***
B.1 vs Uninf.	-	-	-	-	-	**	****	****
β vs δ	-	-	-	0.056	-	-	-	
β vs o	-	-	-	**	***	****	****	
β vs Uninf.	-	-	-	****	****	****	****	
δ vs o	-	-	-	-	-	*	**	***
δ vs Uninf.	-	-	-	-	**	***	****	****
o vs Uninf.	-	-	-	-	-	-	*	*
